# The Human-Associated Archaeon *Methanosphaera stadtmanae* Is Recognized through Its RNA and Induces TLR8-Dependent NLRP3 Inflammasome Activation

**DOI:** 10.3389/fimmu.2017.01535

**Published:** 2017-11-13

**Authors:** Tim Vierbuchen, Corinna Bang, Hanna Rosigkeit, Ruth A. Schmitz, Holger Heine

**Affiliations:** ^1^Division of Innate Immunity, Research Center Borstel, Borstel, Germany; ^2^Institute for General Microbiology, Christian-Albrechts-University Kiel, Kiel, Germany; ^3^Airway Research Center North (ARCN), German Center for Lung Research (DZL), Borstel, Germany

**Keywords:** archaea, *Methanosphaera stadtmanae*, innate immunity, inflammasome, TLR8, NLRP3, monocytes

## Abstract

The archaeon *Methanosphaera stadtmanae* is a member of the gut microbiota; yet, the molecular cross-talk between archaea and the human immune system and its potential contribution to inflammatory diseases has not been evaluated. Although archaea are as bacteria prokaryotes, they form a distinct domain having unique features such as different cell wall structures and membrane lipids. So far, no microbe-associated molecular patterns of archaea which activate innate immune receptors have been identified. By stimulating human myeloid cells with *M. stadtmanae* and purified archaeal nucleic acids, we identified both the microorganism and its RNA as potent stimuli for the innate immune system. To dissect the recognition and activation pathways induced by *M. stadtmanae*, human monocytic BLaER1 knockout cells were generated using the CRISPR/Cas9 system targeting components of TLR and inflammasome signaling. While the recognition of *M. stadtmanae* is mediated by TLR7 and TLR8, activation of the NLRP3 inflammasome depends solely on TLR8 engagement. Notably, this process resembles hallmarks of both the canonical and the recently described alternative inflammasome activation. Thus, we have demonstrated for the first time the specific recognition of and response to an archaeon by human cells at the molecular level.

## Introduction

During the last decade, the extensive use of modern molecular approaches has revealed the existence of trillions of microorganisms in the human intestine that form a complex ecological community (1, 2). Although these microorganisms interact closely with their human hosts to provide many physiological benefits, this community has also been implicated in the development of an increasing number of diseases—in particular, those associated with chronic inflammation (3). Although much research has focused upon bacterial species (which dominate the human gut microbiota), species of the archaeal domain are also stable components of the gut microbiota (4–6). Relatively little is known as to how these less-studied organisms influence human health, although several studies have suggested that methanogenic species of archaea, such as *Methanosphaera stadtmanae*, might be involved in the development of systemic diseases such as obesity (7–10), cancer (11–13), lung hyperresponsiveness (14), and inflammatory bowel disease (IBD) (15). Understanding the molecular mechanisms through which these microorganisms induce inflammation is thus an important step in uncovering how such diseases might develop.

*Methanosphaera stadtmanae* is currently known to be the second most abundant archaeon in the human intestine (16, 17). During the last few years, we and others have demonstrated the high immunogenic potential of *M. stadtmanae* in human peripheral blood mononuclear cells (PBMCs) and monocyte-derived dendritic cells (moDCs) (15, 18, 19)—strong innate and adaptive immune responses, including the secretion of pro-inflammatory cytokines tumor necrosis factor-alpha (TNF-α) and interleukin-1 beta (IL-1β), were detected. Although neither the pattern recognition receptors (PRRs) nor the respective microbe-associated molecular patterns (MAMPs) involved in the response to *M. stadtmanae* have yet been identified, we showed previously that phagocytosis and endosomal acidification are required for recognition of *M. stadtmanae* and resulting cytokine release by both human PBMCs and moDCs (19). Interleukin-1 beta secretion, as seen following exposure to *M. stadtmanae*, results from activation of the inflammasome, a cytosolic multiprotein complex required for processing pro-IL-1β (20). In general, inflammasomes consist of a cytosolic sensor protein, the adaptor protein ASC, and the effector caspase-1. This sensor protein can be a member of the NOD-like receptor (NLR) family or AIM2, a cytosolic DNA sensor (21). NLRP3 is the best-studied member of the NLR family and senses cellular stress signals like potassium efflux and lysosomal leakage (22).

Endosomal recognition of microorganisms often shows an antiviral-type response that is induced through the detection of nucleic acids and includes type-I interferons (IFN-α/β) (23). As a result, we hypothesized that intracellular PRRs might recognize archaeal cellular structures, in particular, their nucleic acids and potentially leading to inflammasome activation. Various cytosolic or endosomal receptors have been identified that are capable of sensing nucleic acids of bacterial or viral origin (24) and might also be involved in the detection of *M. stadtmanae*—for example, toll-like receptors (TLRs), nucleotide-binding oligomerization domain receptors (NLRs), and retinoic acid-inducible gene 1-like receptors (RLRs). However, receptor silencing and inhibition using RNAi constructs or antagonists are often challenging and inefficient in primary human immune cells (25), and this makes it difficult to identify which of these receptors might recognize such archaea. We therefore adopted a loss-of-function approach using CRISPR/Cas9-mediated mutagenesis to obtain stable human knockout cells with a particular focus on intracellular receptors and signaling molecules.

Through this approach, we identified archaeal RNA as the pivotal MAMP of *M. stadtmanae* that activates TLR8 and, to a lesser extent, TLR7. Moreover, *M. stadtmanae* triggers a so far undescribed TLR8-dependent NLRP3 inflammasome activation pathway in human monocytes that shares elements of canonical and alternative inflammasome activation. Thus, our findings describe in detail the molecular mechanisms by which *M. stadtmanae* induces inflammatory responses in human monocytes, which will provide the first steps toward understanding how archaea interact with their host in the gut microbiota and elucidating the potential role of these microorganisms in inflammatory diseases such as IBD or lung hyperresponsiveness.

## Results

### *M. stadtmanae* RNA is a MAMP Inducing an Antiviral Type-I/III Interferon Response in Human Monocytes

**Figure 1:**
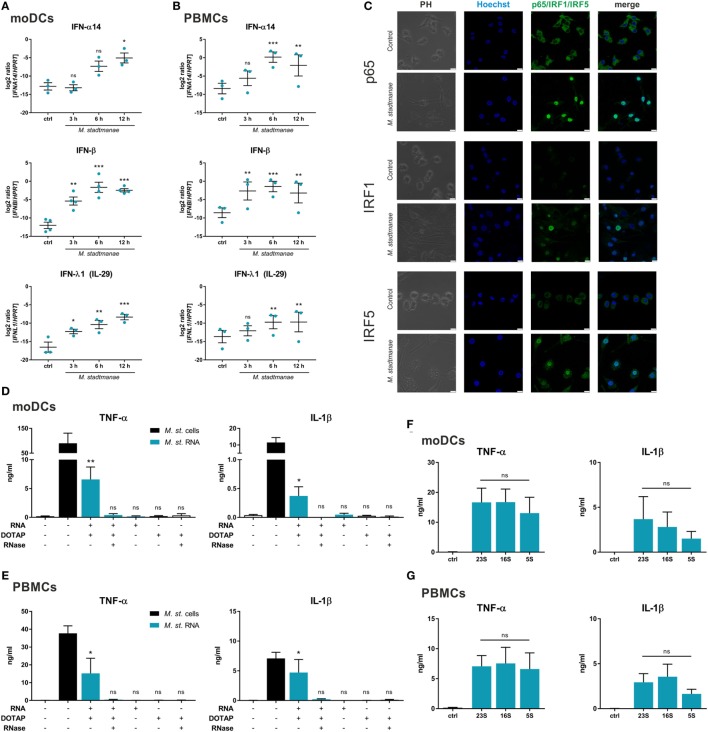
*Methanosphaera stadtmanae* and its RNA inducing an immune response with antiviral characteristics. **(A,B)** The expression of genes encoding for IFN-α14, IFN-β, and IFN-λ1 in moDCs **(A)** and PBMCs **(B)** after stimulation with *M. stadtmanae* for 3, 6, and 12 h was measured by qRT-PCR. The log2 ratios of all three genes to the reference gene *HPRT* are presented. The data from at least three different donors are shown as the mean ± SEM (*n* = 3–4). ns, not significant; *P ≤ 0.05, **P ≤ 0.01, and ***P ≤ 0.001 (all compared with unstimulated control group; repeated measures one-way ANOVA with Dunnett’s *post hoc* test). **(C)** Confocal microscopy of cellular location of NF-κB p65, IRF1, and IRF5 (green) in moDCs after stimulation with *M. stadtmanae* for 4 h by immunolabelling. Nuclei were counterstained with Hoechst 33342 (blue). Scale bars: 10 µM. The images shown are representative examples from one of three independent experiments (*n* = 3). **(D,E)** ELISA of TNF-α and IL-1β in the supernatants of stimulated moDCs **(D)** or PBMCs **(E)** after 18 h. Cells were either untreated, treated with 10^7^ cells of *M. stadtmanae*, or with 5 µg/mL of total RNA from *M. stadtmanae*. RNA was complexed to DOTAP and pre-treated for 30 min at 37°C with RNase A where indicated. **(F,G)** ELISA of TNF-α and IL-1β in the supernatants of moDCs **(F)** or PBMCs **(G)** stimulated for 18 h with 10^7^ cells of *M. stadtmanae* or 2.5 µg/mL of purified rRNAs (complexed to DOTAP). In **(D–G)**, the data shown are the mean ± SEM of at least four different donors (*n* = 4–7). ns, not significant; *P ≤ 0.05 and **P ≤ 0.01 (one-way ANOVA with Tukey *post hoc* test; in **(D,E)** all compared with unstimulated control group and in **(F,G)** the rRNA fractions are compared with each other). moDCs, monocyte-derived dendritic cells; PBMCs, peripheral blood mononuclear cells; qRT-PCR, quantitative reverse-transcription polymerase chain reaction; ANOVA, analysis of variance; TNF-α, tumor necrosis factor-alpha; IL-1β, interleukin-1 beta.

The aim of this study was to elucidate the cellular receptors and MAMPs that are involved in sensing the methanogenic archaeon—*M. stadtmanae* (15, 26). Based on our hypothesis that intracellular PRRs and nucleic acids might be involved, recognition of *M. stadtmanae* should lead to an antiviral-type cellular immune response. Thus, we first analyzed the time-dependent mRNA expression of type-I and type-III IFNs in moDCs (Figure 1A) and PBMCs (Figure 1B). Expression of the genes encoding IFN-α14, IFN-β, and IFN-λ1 (IL-29) was upregulated in PBMCs and moDCs upon exposure to *M. stadtmanae*. As NF-κB, IRF1, and IRF5 are reported to be involved in the response against pathogens and particularly IRF5 in the induction of type-I and type-III IFN responses (27), we next examined the subcellular localization and expression of these transcription factors following cellular activation by *M. stadtmanae*. Using confocal microscopy, we observed that 4 h after stimulation, all three transcription factors were translocated from the cytoplasm into the nucleus (Figure 1C). These findings indicated that these transcription factors were active, and provided further evidence that exposure to *M. stadtmanae* induces an antiviral type-I/III IFN response.

As such immune responses are often induced in response to viral or bacterial nucleic acids, either detected in the cytosol or endocytic compartments, and given that the response to *M. stadtmanae* is also dependent on phagocytosis (19), we hypothesized that the nucleic acids from *M. stadtmanae* might similarly act as MAMPs and activate immune cells. To determine the immunogenicity of archaeal nucleic acids, we transfected purified DNA or RNA into PBMCs using the liposomal transfection reagent DOTAP, and analyzed the secretion of two pro-inflammatory cytokines, TNF-α and IL-1β. Transfection of archaeal DNA did not induce secretion of TNF-α or IL-1β in human PBMCs (Figure S1 in Supplementary Material). In contrast, RNA from *M. stadtmanae* induced TNF-α and IL-1β release from moDCs (Figure 1D), as well as PBMCs (Figure 1E), which was absent upon addition of RNase A to the transfection mix. To analyze if the RNA-dependent activity is restricted to certain RNA species, we purified single ribosomal 5S, 16S, and 23S RNA and examined their capability to activate moDCs (Figure 1F) and PBMCs (Figure 1G). In this experimental setup, no significant changes in cytokine secretion by the different rRNA fractions were observed.

### *M. stadtmanae* is Recognized by Innate Immune Cells through TLR7 and TLR8

Having demonstrated that RNA from *M. stadtmanae* is a potent activator of human immune cells, our next step was to identify the cognate PRR involved in its recognition by human monocytes. Since knockdown approaches in primary human monocytes and dendritic cells were unable to achieve a complete inhibition of gene expression (data not shown), we decided to instead use the BLaER1 cell line (28), in which genetic modifications and stable knock-out *via* CRISPR/Cas9 are possible (29). This line is a B-ALL-derived cell line that can be transdifferentiated into a monocyte-like cell type, so we first confirmed whether these BLaER1 cells, upon differentiation, respond to *M. stadtmanae* in a similar manner to PBMCs and moDCs, and thus whether they would provide a suitable model in which to unpick the molecular mechanisms involved in recognition of this archaeon. Exposure of BLaER1 monocytes to *M. stadtmanae* led to an increase in TNF-α, IL-6, and IL-1β secretion 18 h after stimulation, in a similar manner to that observed with moDCs. Using Cytochalasin D, an inhibitor of phagocytosis, and Bafilomycin A1, an inhibitor of endosomal acidification, we confirmed that the recognition of *M. stadtmanae* in BLaER1 monocytes is also dependent on both mechanisms (Figure S2A in Supplementary Material), similar to our findings in human moDCs (19). The phagocytosis of *M. stadtmanae* by BLaER1 monocytes was verified by confocal microscopy (Figure S2B in Supplementary Material). These data confirmed the use of BLaER1 monocytes as a suitable model system for this study.

**Figure 2:**
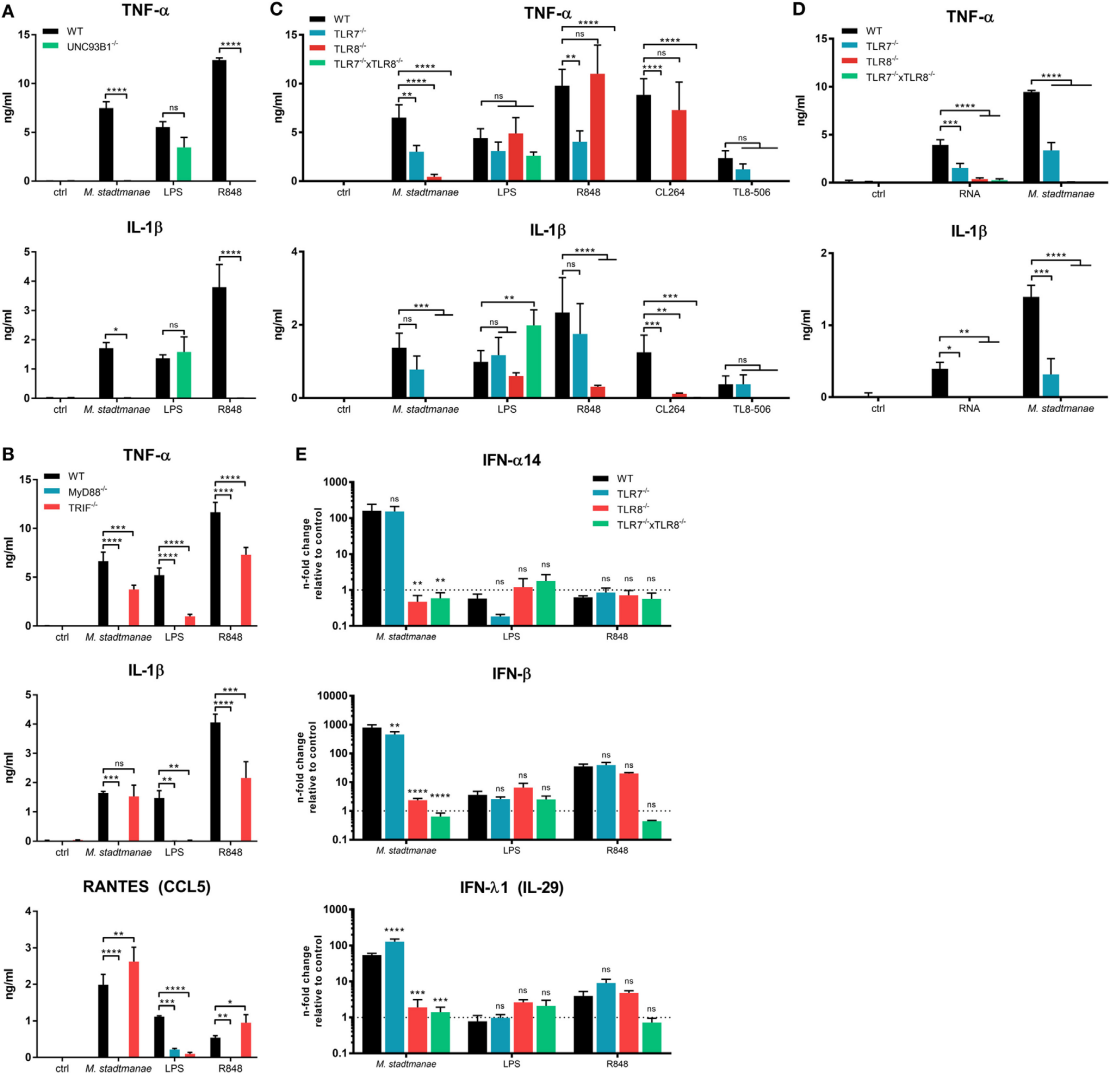
*Methanosphaera stadtmanae* inducing cytokine responses in BLaER1 monocytes through TLR7 and TLR8. **(A)** Secretion of TNF-α, IL-1β, and RANTES (CCL5) in the supernatants of BLaER1 WT and UNC93B1^−/−^ monocytes as measured by ELISA. Cells were stimulated with 10^7^ cells of *M. stadtmanae*, 50 ng/mL LPS or 5 µg/mL R848 for 18 h. **(B)** Secretion of TNF-α and IL-1β in the supernatants of BLaER1 WT and MyD88^−/−^ or TRIF^−/−^ monocytes stimulated as in **(A)**. **(C)** TNF=α and IL-1β secretion in the supernatants of BLaER1 WT, TLR7^−/−^, TLR8^−/−^, or TLR7^−/−^ × TLR8^−/−^ monocytes stimulated for 18 h with either 10^7^ cells of *M. stadtmanae*, 50 ng/mL LPS, or 5 µg/mL of either R848, CL264, and TL8–506. **(D)** Secretion of TNF-α and IL-1β secretion in the supernatants of BLaER1 WT, TLR7^−/−^, TLR8^−/−^, or TLR7^−/−^ × TLR8^−/−^ monocytes stimulated for 18 h with 10^7^ cells of *M. stadtmanae* or 5 µg/mL RNA purified from *M. stadtmanae* and complexed to DOTAP. In **(A–D)**, ns, not significant; *P ≤ 0.05, **P ≤ 0.01, ***P ≤ 0.001, and ****P ≤ 0.0001 (repeated measures two-way ANOVA with Bonferroni *post hoc* test). The data from one representative clone of two are shown as the mean ± SEM of at least three independent experiments (*n* = 3–4). **(E)** The expression of genes encoding IFN-α14, IFN-β, and IFN-λ1 in BLaER1 WT and TLR7^−/−^, TLR8^−/−^ or TLR7^−/−^ × TLR8^−/−^ monocytes after stimulation with *M. stadtmanae* (100:1), 50 ng/mL LPS, or 5 µg/mL R848 for 8 h was measured by qRT-PCR. The expression ratios of all three IFN genes to the reference gene *HPRT* are shown as *n*-fold induction relative to the unstimulated control. ns, not significant; **P ≤ 0.01, ***P ≤ 0.001, and ****P ≤ 0.0001 [repeated measures two-way ANOVA with Bonferroni *post hoc* test in **(A)** and Dunnett’s *post hoc* test in **(B–E)**]. The data are shown as the mean ± SEM of three independent experiments (*n* = 3). qRT-PCR, quantitative reverse-transcription polymerase chain reaction; ANOVA, analysis of variance.

Our data suggest that *M. stadtmanae* RNA is detected by intracellular PRRs in human monocytes. All human nucleic acid-specific TLRs (TLR3 and TLR7-9) are located in the endosomal compartment, and these receptors rely on the protein UNC93B1, which is mandatory for their trafficking from the endoplasmic reticulum (ER) to the endosomes (30). To confirm our assumption that *M. stadtmanae* is recognized by endosomal TLRs, we generated UNC93B1^−/−^ cells (see Figures S3A,B in Supplementary Material for further information regarding all CRISP/Cas9-generated BLaER1-KO cells in this study) and determined whether these cells retained an ability to respond upon exposure to archaeal cells. UNC93B1^−/−^ BLaER1 monocytes failed to secrete any TNF-α and IL-1β after stimulation with *M. stadtmanae* (Figure 2A), confirming the involvement of either TLR3, or TLR7-9 in the induction of cytokine responses.

We next generated KO cells lacking MyD88 or TRIF, which are the central adapter molecules used by different TLRs for signal transduction (31). Upon exposure to *M. stadtmanae*, TRIF^−/−^ cells showed a slightly decreased but still high secretion of TNF-α and no reduction in the levels of IL-1β and RANTES (CCL5), compared with that induced in stimulated WT cells (Figure 2B). In contrast, this response was completely absent in MyD88^−/−^ cells exposed to this archaeal species (Figure 2B). Our results showed that *M. stadtmanae* is recognized through one or more MyD88-dependent TLRs by human monocytes.

As TLR9 recognizes DNA, TLR3 signals *via* TRIF, and human TLR7 and TLR8 are the only RNA-specific receptors that signal *via* MyD88 and are dependent on UNC93B1, we assumed that either or both of TLR7 and TLR8 were involved in the detection of *M. stadtmanae*. To identify which of these receptors recognizes *M. stadtmanae*, we generated single-gene knockout cell lines for each these receptors, as well as a double knockout (TLR7^−/−^ × TLR8^−/−^) cell lines. Using these cells, we demonstrated that the secretion of TNF-α in response to *M. stadtmanae* can be induced *via* TLR7 as well as TLR8, with reduced levels of cytokine secretion detected compared with that produced by stimulated WT cells in both knockout cell lines. TLR8^−/−^ cells did, however, show a greater decrease in TNF-α secretion compared with that of the TLR7^−/−^ cells (Figure 2C). Unexpectedly, the secretion of IL-1β was entirely dependent on TLR8, as no secretion of this cytokine was detected in TLR8^−/−^ cells after stimulation with *M. stadtmanae*. The TLR7/8 agonist R848, the TLR7 agonist CL264, and the TLR8 agonist TL8–506 were used as controls to confirm the specificity of the clones used in this study (Figure 2C). Additionally, we transfected purified RNA from *M. stadtmanae* into TLR7^−/−^, TLR8^−/−^, and TLR7^−/−^ × TLR8^−/−^ BLaER1 monocytes using DOTAP and measured the secretion of TNF-α and IL-1β after 18 h. Similar to whole archaea, RNA from *M. stadtmanae* is recognized by TLR7 and TLR8, but the knockout of TLR8 revealed that the latter receptor has a stronger effect (Figure 2D).

Recently, TREML4 was shown to be involved in the recruitment of MyD88 to TLR7, thereby amplifying its signaling (32). However, TREML4^−/−^ BLaER1 monocytes showed no alterations in secretion of IL-6 or TNF-α after stimulation with *M. stadtmanae* or different TLR7/8 agonists (Figure S4 in Supplementary Material) indicating that this protein, at least in human monocytes, is not essential for TLR7/8 activation. To further characterize the individual roles of TLR7 and TLR8 in the response to *M. stadtmanae*, we used quantitative reverse-transcription polymerase chain reaction (qRT-PCR) to analyze the expression of type-I and type-III IFN mRNAs in the different TLR-KO cell lines after 8 h stimulation with *M. stadtmanae*. In TLR7^−/−^ cells, only IFN-β showed a slight reduction in expression compared with the levels in WT stimulated monocytes (Figure 2E), whereas expression of genes encoding all three IFNs, IFN-α14, IFN-β, and IFN-λ1, were significantly reduced from WT levels in TLR8^−/−^ cells, and an even stronger effect was detected in double-KO cells (Figure 2E). These data demonstrated that not only pro-inflammatory cytokines such as TNF-α and IL-1β but also type-I/III IFNs are induced by activation of TLR8 (and to a minor degree TLR7) with *M. stadtmanae*.

As mice and humans differ in their repertoire of RNA-specific TLRs (33), we also analyzed the cytokine response of bone marrow-derived dendritic cells (BMDCs) from different knockout mice after stimulation with *M. stadtmanae* (Figure S5 in Supplementary Material). Using these cells, we demonstrated that in mice, the recognition of this archaeon is completely dependent on mTLR7, whereas the absence of mTLR8 or mTLR13 had no effect on the secretion of IL-6.

### *M. stadtmanae* Inducing the Secretion of IL-1β *via* Activation of the NLRP3 Inflammasome

**Figure 3:**
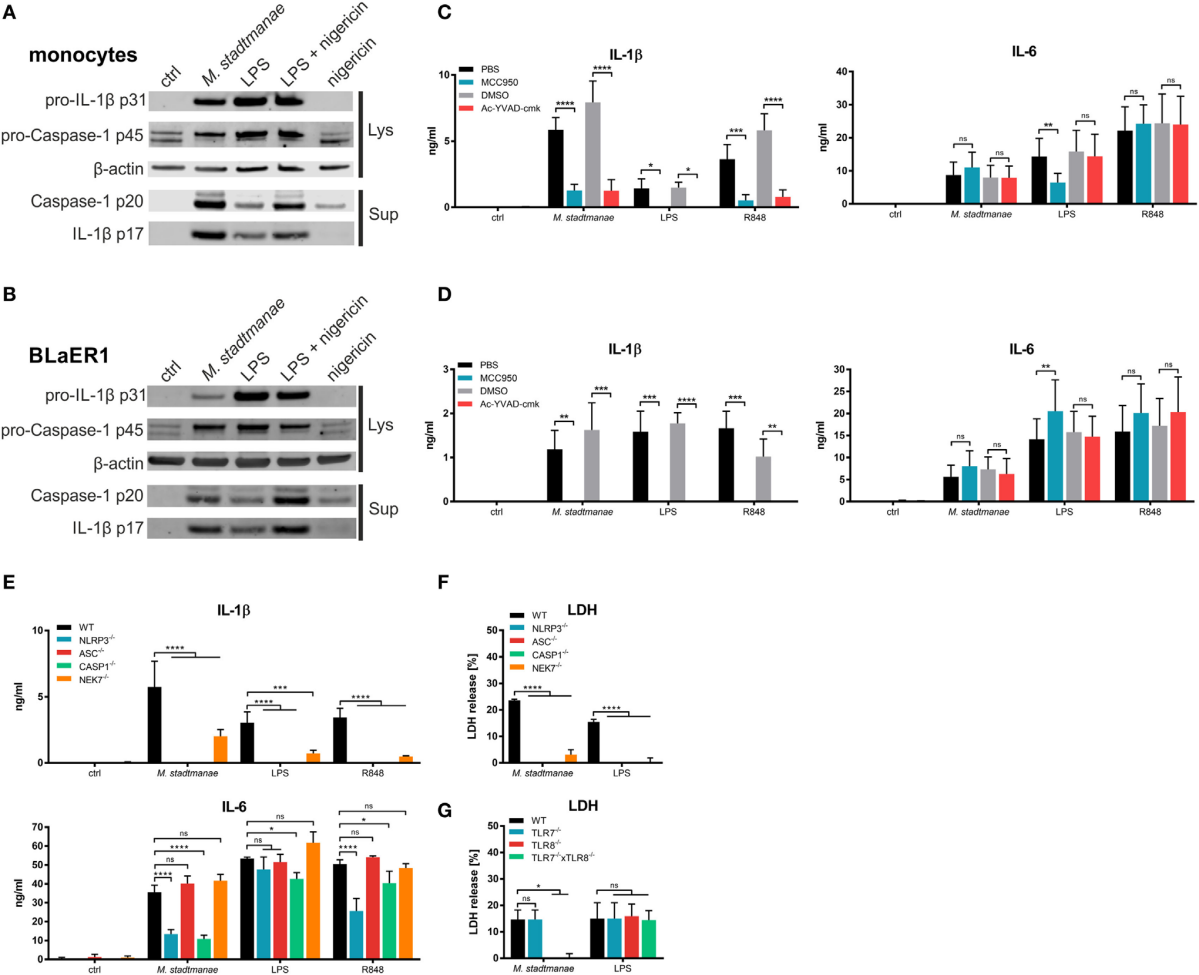
*Methanosphaera stadtmanae* activating the NLRP3 inflammasome in primary and BLaER1 monocytes. **(A,B)** Immunoblotting of cell lysates and supernatants of primary monocytes **(A)** and BLaER1 WT monocytes **(B)** showing the activation status of caspase-1 (p20) and IL-1β (p17). Additionally, the pro-forms of both proteins, namely caspase-1 p45 and IL-1β p31 were analyzed in the cell lysate. Analysis of β-actin was used as loading control. Cells were stimulated with *M. stadtmanae* (100:1) or 50 ng/mL LPS for 18 h. 13.4 µM nigericin was added where indicated for the last 2 h. Blots are shown either from one representative donor of three in **(A)** or as one representative of three independent experiments in **(B)** (*n* = 3). **(C,D)** The secretion of IL–6 and IL–1β in the supernatants of stimulated primary monocytes **(C)** or BLaER1 WT monocytes **(D)** as measured using ELISA. Inhibitors of NLRP3 and caspase-1 (MCC950 and Ac-YVAD-cmk, respectively) and control substances (medium and DMSO, respectively) were added 1 h before stimuli were added. Cells were stimulated with 10^7^ cells of *M. stadtmanae*, 50 ng/mL LPS, or 5 µg/mL R848 for 18 h. ns, not significant; *P ≤ 0.05, **P ≤ 0.01, ***P ≤ 0.001, and ****P ≤ 0.0001 (repeated measures two-way ANOVA with Tukey *post hoc* test). The data from at least three different donors in **(C)** (*n* = 3–4) or three independent experiments in **(D)** (*n* = 3) are shown as the mean ± SEM. **(E)** The secretion of IL-6 and IL-1β in the supernatants of stimulated primary monocytes or BLaER1 WT and KO monocytes (as indicated in the figure) was measured using ELISA. Cells were stimulated with 10^7^ cells of *M. stadtmanae*, 50 ng/mL LPS, or 5 µg/mL R848 for 18 h. ns, not significant; *P ≤ 0.05, ***P ≤ 0.001, and ****P ≤ 0.0001 (repeated measures two-way ANOVA with Dunnett’s *post hoc* test). The data from one representative clone of two (except for NLRP3) of three independent experiments (*n* = 3) are shown as the mean ± SEM. **(F,G)** Detection of LDH in the supernatant of stimulated BLaER1 WT and KO monocytes (as indicated in the figure). Cells were stimulated with 10^7^ cells of *M. stadtmanae*, 50 ng/mL LPS, or 5 µg/mL R848. ns, not significant; *P ≤ 0.05 and ****P ≤ 0.0001 (repeated measures two-way ANOVA with Dunnett’s *post hoc* test). The data from one representative clone of two (except for NLRP3) of three independent experiments (*n* = 3) are shown as the mean ± SEM. ANOVA, analysis of variance; LDH, lactate dehydrogenase.

Maturation and release of IL-1β along with that of the pro-inflammatory cytokine IL-18, occurs upon activation of caspase-1, following activation of the inflammasome and recruitment of apoptosis-associated speck-like protein containing a CARD (ASC) in response to PRR signaling. Inflammasomes are cytosolic multiprotein complexes that differ in their utilization of receptor proteins such as NLRP3 or AIM2 (20). To clarify the process of IL-1β release and inflammasome activation induced by *M. stadtmanae*, we first analyzed the release of active caspase-1 p20 and mature IL-1β p17 in the supernatant of human monocytes using immunoblotting. After stimulation for 18 h with *M. stadtmanae*, caspase-1 p20 and IL-1β p17 were detected in the supernatant of both primary human monocytes (Figure 3A) and BLaER1 monocytes (Figure 3B), indicative of inflammasome activation in both these cell types. To identify the type of inflammasome that is activated by *M. stadtmanae*, we first determined the effects of specific inhibitors for caspase-1 (Ac-YVAD-cmk) and NLRP3 (MCC950) (34) upon the induction of pro-inflammatory cytokine responses. Both inhibitors almost completely abolished the release of IL-1β observed in untreated primary human monocytes (Figure 3C) and BLaER1 monocytes (Figure 3D) after stimulation with *M. stadtmanae*, although release of IL–6 remained unaffected by either inhibitor confirming their specificity. These findings demonstrated the dependency of *M. stadtmanae*-induced inflammasome activation on caspase-1 and NLRP3. An equivalent observation was made for primary moDCs and PBMCs (Figures S6A,B in Supplementary Material).

To confirm activation of the NLRP3 inflammasome by *M. stadtmanae*, we generated additional BLaER1 cell lines in which NLRP3, ASC, or caspase-1 were knocked out, and stimulated these cells with *M. stadtmanae* (Figure 3E). The results were consistent with those obtained using inhibitors, showing that all three molecules were required for the secretion of IL-1β. However, NLRP3^−/−^ and caspase-1^−/−^, but not ASC^−/−^, cells also showed a decrease in IL-6 secretion after stimulation with *M. stadtmanae*. In addition, we tested the involvement of NEK7, a molecule that was recently shown to be required for NLRP3 inflammasome activation (35). BLaER1 NEK7^−/−^ cells also displayed a significant decrease in IL-1β secretion (Figure 3E), but the effect was not as strong as that in the NLRP3, ASC, and caspase-1 knockouts.

Pyroptosis, an inflammatory form of cell death, has been reported to follow NLRP3 inflammasome activation, and to be involved in the release of pro-inflammatory cytokines (20). To test whether pyroptosis was a consequence of *M. stadtmanae*-induced NLRP3 activation, cell death was measured by detecting the release of lactate dehydrogenase (LDH) from stimulated and unstimulated BLaER1 WT and KO monocytes. BLaER1 WT cells showed a release of approximately 20% of the amount of LDH released by the cell lysis control after stimulation with *M. stadtmanae*. By contrast, LDH release, and thus cell death, was undetectable in cells lacking the inflammasome components NLRP3, ASC, caspase-1, or NEK7 (Figure 3F), and was also absent from TLR8^−/−^ and TLR7^−/−^ × TLR8^−/−^ cells (Figure 3G) indicating that activation of TLR8 by *M. stadtmanae* hence leads to induction of pyroptosis.

### *M. stadtmanae* Inducing the Secretion of IL-1β without the Formation of ASC Specks in BLaER1 Monocytes

**Figure 4:**
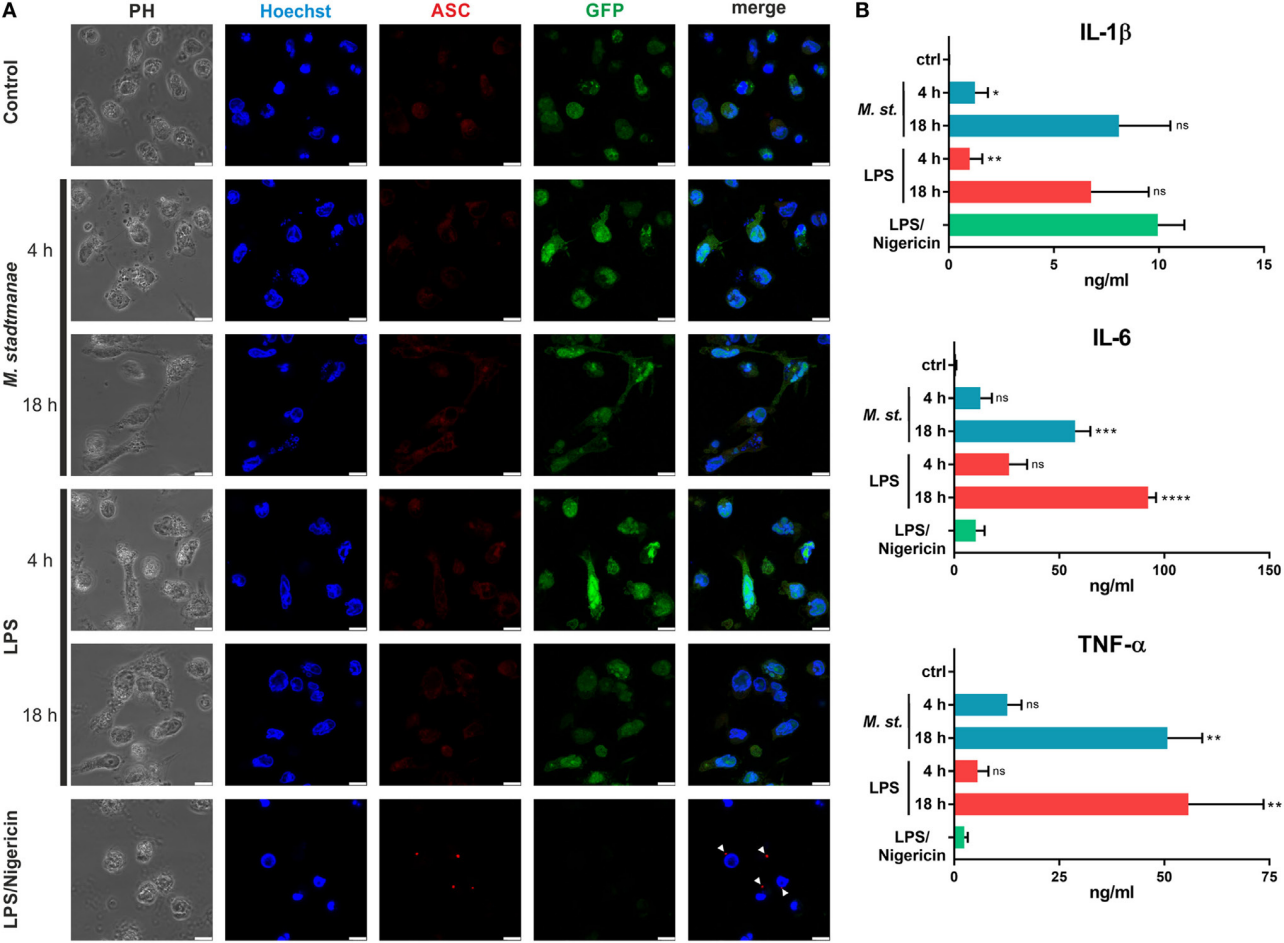
*Methanosphaera stadtmanae* not inducing formation of ASC specks in BLaER1 monocytes. **(A)** Confocal microscopy was used to visualize fixed BLaER1 monocytes stained with ASC-specific antibodies (red). Cells were stimulated for 4 or 18 h with 10^7^ cells of *M. stadtmanae* or 100 ng/mL LPS. LPS/nigericin stimulation was performed for 4 h, with 13.4 µM nigericin being added 3 h after initial LPS stimulation. After fixation, cells were stained with anti-ASC antibodies. DNA was stained with Hoechst 33342 (blue), and cytoplasmic GFP expression is shown in green. White arrowheads depict ASC specks. Scale bars: 10 µM. The data are shown from one representative of three independent experiments (*n* = 3). **(B)** The secretion of IL-1β, IL-6, and TNF-α from cells shown in **(A)** was measured using ELISA. Values are compared with those obtained using LPS/nigericin stimulation. ns, not significant; *P ≤ 0.05, **P ≤ 0.01, ***P ≤ 0.001, and ****P ≤ 0.0001 (one-way ANOVA with Dunnett’s *post hoc* test). The data are from three independent experiments (*n* = 3) and shown as the mean ± SEM. ANOVA, analysis of variance.

Several pathways for inflammasome activation have previously been described, including canonical, non-canonical, and alternative activation. They differ in terms of the stimuli responsible, and the formation of ASC specks: in particular, alternative activation in response to LPS stimulation and TLR4 signaling was recently shown to be independent of ASC speck formation (29) otherwise characteristic of both canonical and non-canonical inflammasome activation. To distinguish which of these activation pathways was induced upon stimulation of human monocytes by *M. stadtmanae*, we examined whether ASC specks could be observed in these cells. As expected, ASC speck formation was detectable in control BLaER1 cells treated with LPS and nigericin for 4 h to induce canonical inflammasome activation (Figure 4A). Conversely, in cells treated with LPS alone, which induces the alternative activation pathway, ASC specks were absent both 4 and 18 h after treatment (Figure 4A). Although we were able to show TLR8-dependent induction of pyroptosis in BLaER1 monocytes, usually a hallmark of canonical activation, cells stimulated with *M. stadtmanae* showed no ASC speck formation, a phenotype more comparable to that of cells stimulated with LPS alone and alternative activation. Secretion of IL-1β in BLaER1 monocytes after 4 h with *M. stadtmanae* or LPS alone was relatively weak (Figure 4B), however, by 18 h, the level of IL-1β secreted by these cells was much higher and comparable to that induced by canonical inflammasome activation, despite the lack of formation of ASC specks. Taken together, these results demonstrated that *M. stadtmanae* triggers a TLR8-dependent NLRP3 inflammasome activation pathway in human monocytes that shares characteristics of canonical as well as alternative inflammasome activation.

## Discussion

Only recently have researchers begun to appreciate the full diversity of our microbiome and to include genetic data from other domains (17). Due to methodological challenges (36), the function and influence of archaea on the human immune system have yet to be determined and, the exact mechanism by which *M. stadtmanae* induces inflammation remains unknown. Although much is known of the molecular interactions involved in bacterial-induced inflammation, these findings are not immediately applicable to the detection of archaea by immune cells: bacteria and archaea are genetically and structurally different. In particular, the various archaeal cell wall structures and membrane lipids are very different from those of bacteria [e.g., the methanoarchaea *M. stadtmanae* and *Methanobrevibacter smithii* do not engage NOD2, TLR2, or TLR4 (19)], and their translation and transcription machineries resemble eukaryotic processes (26). Here, we describe for the first time the key archaeal MAMP, in the form of *M. stadtmanae* RNA which is recognized by human immune cells through TLR8 and, to a lesser extent, through TLR7.

We consider activation of human myeloid cells by *M. stadtmanae* to be reminiscent of antiviral characteristics, as it induces the gene expression of type-I and type-III IFNs and the translocation of IRF1 and IRF5 into the nucleus. The cytokine response is completely dependent on MyD88 and UNC93B1 but not on TRIF—these findings indicate the involvement of nucleic acids, specifically ssRNA, in the recognition process. We clearly demonstrate that archaeal RNA, but not DNA, can induce a pro-inflammatory response. Recently, there has been great interest in bacterial RNA as a potent MAMP sensed by TLR7 (37, 38) and TLR8 (39, 40) in human immune cells. The activation of these receptors through bacterial RNA leads to secretion of pro-inflammatory cytokines and type-I IFNs, such as IFN-α and IFN-β (23). Archaeal RNA activates immune cells in a similar manner and irrespective of the rRNA species. This observation is consistent with the recently unraveled crystal structures and binding mechanisms of TLR7 (41) and TLR8 (42), as both receptors bind to the degradation products of RNA. In contrast to murine TLR13, which binds to a specific sequence in bacterial 23S rRNA (43), the activation of TLR7 and TLR8 is rather sequence-independent. Both receptors concurrently recognize single nucleotides and short oligonucleotides (2–3mers), which should be present in all fractions of RNA from *M. stadtmanae*. This mechanism appears to be promiscuous and capable of recognizing a wide range of organisms; thus, it is not restricted to bacteria but likely involved in the recognition of viruses, and presumably other archaeal species besides *M. stadtmanae*. In BLaER1 monocytes, the recognition of *M. stadtmanae* is mediated primarily by TLR8 and to a lesser extent by TLR7. Notably, the induction of IFN-α14 and IFN-λ1 as well as the secretion of IL-1β is fully dependent on TLR8. This difference in receptor involvement might be due to the unequal expression of the two receptors, as TLR8 rather than TLR7 seems to be the predominant receptor in human monocytes (44). Additionally, TLR8 and TLR9 seem to outperform TLR7 in terms of UNC93B1-mediated trafficking to the endosomes (45), which may explain the stronger influence of TLR8 on *M. stadtmanae-*induced secretion of TNF-α and IL-6.

We demonstrated that in addition to inducing pro-inflammatory cytokines and IFNs, *M. stadtmanae* also potently activates the NLRP3 inflammasome. As secretion of IL-1β is completely lost in NLRP3^−/−^ cells, it clearly indicates that the NLRP3 inflammasome is the essential executor activated by *M. stadtmanae*. The contribution of other inflammasome complexes (e.g., in priming steps), however, cannot be completely ruled out. With the use of inhibitors and BLaER1 KO monocytes, we revealed that inflammasome activation is TLR8-dependent and RNA-mediated. This process is most likely related to the activation process induced by bacterial RNA, which was also shown to trigger the NLRP3 inflammasome, leading to caspase-1 activation and the conversion of pro-IL–1β into its active form (46, 47). However, the process of *M. stadtmanae*-induced inflammasome activation differs from classical activation pathways, namely, canonical and non-canonical activation, which both lead to the formation of ASC specks and cell death after short stimulation times (48). In the case of *M. stadtmanae*, inflammasome activation is delayed compared with classical NLRP3 activation (e.g., by LPS/nigericin stimulation). Furthermore, *M. stadtmanae* did not induce ASC speck formation in BLaER1 monocytes, although IL-1β was clearly present in the supernatant after 18 h. This activation resembles the recently described process of alternative inflammasome activation (29). However, in contrast to this TLR4- and TRIF-dependent alternative activation, *M. stadtmanae* induces TLR8-dependent inflammasome activation and TLR8- and NLRP3-dependent cell death, known as pyroptosis, independently of TRIF. Furthermore, we detected LDH release after stimulation with LPS alone. One possible explanation is that this mechanism is caspase-4-mediated, as Gaidt et al. used caspase-4^−/−^ BLaER1 monocytes as control cells.

Given that TLR8, but not TLR7, is involved in reactive oxygen species (ROS) production by the NADPH oxidase NOX2 complex in human neutrophils (49) and a relationship between the NLRP3 inflammasome and NOX2 was previously demonstrated in relation to phagosomal acidification (50), we hypothesize that *M. stadtmanae*-induced TLR8-dependent NLRP3 activation is associated with the production of ROS (51). ROS production might provide a necessary signal for inflammasome activation, especially as these reactive intermediates can serve as an NEK7-mediated activation signal of the NLRP3 inflammasome (52). Thus, ROS may provide a feasible mechanism promoting *M. stadtmanae*-induced NLRP3 activation. Using BLaER1 NEK7^−/−^ monocytes, we were able to confirm the involvement of NEK7 in *M. stadtmanae*-induced cytokine production, although this phenotypic loss was not as strong as that observed in cells lacking ASC, Caspase-1, NLRP3, or TLR8. As a result, the detailed mechanism of how NEK and ROS influence NLRP3 activation will require further clarification.

The findings regarding the interaction between the methanogenic archaeon *M. stadtmanae* and human immune cells indicate that archaea represent an important part of our microbiota. There is a great need for further investigation of human-associated archaea, if one considers that many inflammatory diseases are associated with both the inflammasome (53, 54) and the composition of the microbiota (3). As we identified *M. stadtmanae* as a strong activator of pro-inflammatory immune responses *in vitro*, it is conceivable that it could also contribute to an over-activation of the inflammasome *in vivo*. Therefore, future studies should adapt their experimental design to consider these important components of our microbiota and better understand the involvement of archaea in the development of inflammatory diseases such as IBD and lung hypersensitivity, and thus shape possible treatment approaches.

## Materials and Methods

### Ethics Approval Statement

Approval for these studies was obtained from the Institutional Ethics Committee at the University of Lübeck (Lübeck, Germany; Protocol no. Az. 12-202A) according to the Declaration of Helsinki. All donors gave written informed consent.

### *M. stadtmanae* Growth and Media

*Methanosphaera stadtmanae* (DSM 3091) was grown as previously described (55). Immune cell stimulation experiments were carried out with exponentially growing *M. stadtmanae* cells that were centrifuged at 3,200× *g* for 30 min, washed, and suspended in aerobic 50 mM Tris–HCl (pH 7.0).

### Isolation of Archaeal RNA and DNA

For the isolation of nucleic acids, *M. stadtmanae* cells were grown as previously described (55), harvested at 4°C (3,200× *g* for 30 min), and lysed in liquid nitrogen using a Mikro-Dismembrator S laboratory ball mill (Sartorius) for 3 min at 1,600 bpm. RNA was isolated by TRIzol extraction followed by DNase I treatment. DNA was isolated using the Wizard Genomic DNA Purification Kit (Promega) according to the manufacturer’s protocol. Concentration and purity were determined using the DS-11 spectrophotometer (DeNovix). RNA with an A_260_/A_280_ ratio ≥ 2.0 was considered as pure RNA; DNA with an A_260_/A_280_ ratio of approximately 1.8 was considered as pure DNA.

The separation of ribosomal RNA fractions (5S, 16S, and 23S rRNA) was carried out by excision of the respective fractions after agarose gel electrophoresis and subsequent purification using NucleoSpin Gel and PCR Clean-up Kit according to the manufacturer’s protocol (Macherey-Nagel).

### Cell Culture

Peripheral blood mononuclear cells were prepared from the heparinized blood of donors by gradient centrifugation (56) using Biocoll (Merck). Subsequently, monocytes were isolated by counter-flow elutriation centrifugation (57). Monocyte-derived dendritic cells were then generated from monocytes as previously described (58). All cells were cultured in RPMI 1640 medium with stable glutamine supplemented with 10% FCS and antibiotics [100 U/mL penicillin and 100 µg/mL streptomycin (P/S; all from Merck)] from now on referred to as complete medium. Cells were grown and incubated in a humidified atmosphere of 5% carbon dioxide at 37°C.

BLaER1 cells were a gift from Thomas Graf (Center for Genomic Regulation, Barcelona, Spain). The cells were cultured under the same conditions as the moDCs and maintained at a cell density between 1 × 10^5^ and 2 × 10^6^ cells/mL. Transdifferentiation was induced by cultivating 3 × 10^5^ cells/mL for 6–7 days in complete medium with 10 ng/mL IL-3, 10 ng/mL M-CSF (both Peprotech), and 100-nM β–estradiol (Sigma), as previously described (28). For stimulation, cells were replated in complete medium without IL-3, M-CSF, or β-estradiol. The process of transdifferentiation was regularly checked using standard flow cytometry analysis with CD19-BV421, CD11b-APC, and CD14-PE antibodies (all from BioLegend) on a MACSQuant Analyzer 10 (Miltenyi Biotec).

### Cell Stimulation

Peripheral blood mononuclear cell and moDC were stimulated for 18 h at a cell density of 1 × 10^5^ cells/well in a 96-well flat-bottom plate with a total volume of 200 µL. Monocytes and BLaER1 cells were stimulated at a cell density of 0.25 × 10^5^ and 0.5 × 10^5^ cells/well, respectively. Whole cells of *M. stadtmanae* were applied at 10^7^ cells per well, unless otherwise indicated. LPS (from *Escherichia coli* O111, a gift from Otto Holst, Research Center Borstel) was added at a final concentration of 50 ng/mL. R848, CL264, and TL8–506 (all from InvivoGen) were used at 5 µg/mL. Archaeal RNA was complexed with the liposomal transfection reagent DOTAP (Carl Roth) prior to stimulation at a ratio of 6.5 µL DOTAP per 1 µg RNA in 50 µL of pure RPMI 1640. Total RNA was added at a concentration of 5 µg/mL, and purified rRNA was added at 2.5 µg/mL. Three hours after the addition of RNA, 50 µL of complete RPMI 1640 was added to the cells. When indicated, RNA was digested using RNase A, DNase I or Proteinase K (Thermo Fisher) prior to complexing with DOTAP as recommended by the manufacturer.

For the inhibition experiments, 7.5 µM MCC950, 50 µM Ac-YVAD-cmk (both Sigma-Aldrich), 2 µM Cytochalasin D (Sigma), or 10 nM Bafilomycin A1 (Merck) was added to cells 1 h prior to stimulation.

### Generation of Stable Knockout Cell Lines Using CRISPR/Cas9

Gene-specific gRNA sequences were designed using Benchling^1^ online software, in which off-target scores were kept as low as possible. The gRNA sequences that were used in this study are listed in Table S1 in Supplementary Material. The BbsI restriction site was used to clone ODNs (Thermo Fisher) encoding gRNA sequences into pU6-(BbsI)-CBh-Cas9-T2A-BFP [a kind gift from Ralf Kuehn (59); Addgene plasmid #64323] as previously described (60). Using the Human B Cell Nucleofector Kit and Nucleofector I device (program U-15; both Lonza), 1 × 10^6^ cells were transfected with 2 µg plasmid DNA. Forty-eight hours after transfection, GFP^+^/BFP^+^ cells were sorted into 96-well plates (1 cell/well) by the Fluorescence Cytometry core unit at the Research Center Borstel using a FACSAria IIu (BD Biosciences). Two to three weeks after sorting, DNA from the clones was isolated using QuickExtract DNA Extraction Solution (Epicenter). Specific gene fragments were amplified using standard PCR methods and sequenced at Eurofins Genomics. The occurrence of InDel mutations was analyzed using the Tracking of Indels by Decomposition (TIDE)^2^ online software (61). Only clones with frameshift mutations on all target alleles were used for further experiments. Clones were characterized using western blot and/or ELISA depending on the availability of specific antibodies. The results of the immunoblot and TIDE analysis are shown in Figures S3A,B in Supplementary Material, respectively.

### Cytokine Measurements and LDH Release Assay

The concentrations of released cytokines in the supernatants were determined after 16 h using commercial ELISA kits specific for hIL-1β, hIL-6, mIL-6, hTNF-α (Thermo Fisher), and hRANTES (CCL5) (R&D Systems). LDH release was measured using the Pierce LDH Cytotoxicity Assay Kit (Thermo Fisher) according to the manufacturer’s instructions. LDH release (%) was calculated as follows: (stimulated sample − unstimulated sample)/(lysis control − unstimulated sample) × 100.

### Quantitative Reverse-Transcription Polymerase Chain Reaction

For expression analysis *via* qRT-PCR, 0.5 × 10^6^ or 1 × 10^6^ PBMCs, moDCs, or BLaER1 monocytes were stimulated for the indicated time periods (3, 6, and 12 h for PBMCs and moDCs; 8 h for BLaER1 monocytes) with *M. stadtmanae* cells (100:1) in a total volume of 0.5 or 1 mL at 37°C. After stimulation, cells were harvested, and RNA was isolated using the NucleoSpin RNA Isolation Kit (Macherey-Nagel) according to the manufacturer’s instructions. The concentration and purity of RNA were analyzed using a DS-11 spectrophotometer (DeNovix). Reverse Transcription of RNA into cDNA was achieved using SuperScript III, RNase Out, dNTPs, and oligo dT primer (all from Thermo Fisher) according to the manufacturer’s instructions. Quantitative PCR was performed using SYBR Green Master Mix on a LightCycler 480 II (both Roche). All primers were purchased from Thermo Fisher, and their sequences are listed in Table S2 in Supplementary Material. The following cycling conditions were applied: 95°C for 10 min (95°C for 10 s; 63 −58°C for 10 s with −0.5°C/cycle and 72°C for 6 s) × 45 cycles. The data were analyzed using LightCycler 480 software (v. 1.5.1) by calculating the ratio of the target gene expression to expression of the reference gene *HPRT*. Data were presented either as log2 ratio or as *n*-fold induction compared with unstimulated cells.

### Immunoblotting

Human monocytes or BLaER1 cells were stimulated with *M. stadtmanae* at a ratio of 1:100 in RPMI 1640 with 1% P/S (both Merck) for 18 h. LPS at 50 ng/mL was used as a control, and 13.4 µM nigericin (Invivogen) was added for the last 2 h. Supernatants were precipitated with trichloroacetic acid (1:10, Sigma), and the pellet was washed three times with acetone and suspended in LDS buffer (Thermo Fisher) with 5% β-mercaptoethanol (Carl Roth). Cell lysates were generated by direct lysis of the cells in LDS buffer with 5% beta-mercaptoethanol. To analyze KO clones, cell lysates from BLaER1 KO monocytes were generated in the same manner. Gel electrophoresis was performed using NuPage Novex 12% Bis-Tris protein gels (Thermo Fisher) for 45 min at 200 V, and proteins were subsequently blotted onto PVDF membranes (Carl Roth) for 1 h at 30 V. Membranes were blocked using Roti-Block (Carl Roth), and primary antibodies were added overnight at 4°C. Caspase-1 p20 (Bally-1, 1:1,000, Adipogen, used for detection of p45 and p20), IL–1β (D3U3E, 1:1,000, used for detection of p31 and p17), β-actin (8H10D10, 1:5,000, both Cell Signaling), ASC (B-3, 1:1,000, Santa Cruz Biotechnology), NLRP3 (Cryo-2, 1:1,000, Adipogen), and NEK7 (EPR4900, 1:5,000, Abcam) were used as primary antibodies. Goat-anti-mouse-Alexa 680 (Thermo Fisher) and goat-anti-rabbit-IRDye 800CW (LI-COR Biosciences) were used as secondary antibodies at 1:10,000 dilution. The blots were visualized with the LI-COR Odyssey system (LI-COR Biosciences).

### Confocal Laser Scanning Microscopy

For confocal laser scanning microscopy, 10^5^ moDC were incubated at 37°C for 2 h on 6 channel μ-Slide (Ibidi) and subsequently stimulated for 4 h with 10^7^ *M. stadtmanae* cells. After fixation in 3% paraformaldehyde, primary antibodies were added at 1:100 in PBS with 3% BSA and 0.1% saponin and incubated overnight at 4°C. Cells were incubated with anti-NF-κB p65 (1:100, F-6), anti-IRF1 (1:100, B-1), or anti-IRF5 (1:100, H-56, all from Santa Cruz Biotechnology) overnight at 4°C, followed by staining with an Alexa Fluor 488-conjugated goat anti-mouse IgG (H + L) secondary antibody (1:300, Invitrogen). The nuclei were counterstained using Hoechst 33342 dye (1:3,000, Life Technologies). For detection of ASC specks, BLaER1 cells were stimulated for 4 and 18 h, and staining was performed as previously described. ASC (1:100, B-3, Santa Cruz Biotechnology) was used as the primary antibody, and Alexa Fluor 546-conjugated goat anti-mouse IgG (H + L) was used as the secondary antibody (1:300, Invitrogen). Images were captured using the TCS SP5 confocal microscope and LAS AF software (both from Leica).

### Statistical Analysis

The data were analyzed for statistical significance using a one-way or two-way ANOVA with a Bonferroni, Dunnett’s or Tukey *post hoc* test using Graph Pad Prism 7.02 software. The values of *P* ≤ 0.05 were considered to be statistically significant.

## Ethics Statement

Approval for these studies was obtained from the Institutional Ethics Committee at the University of Lübeck (Lübeck, Germany; Protocol no. Az. 12-202A) according to the Declaration of Helsinki. All donors gave written informed consent.

## Author Contributions

TV, CB, RAS, and HH designed the research. TV, CB, and HR performed the research. TV, CB, HR, and HH analyzed the data. TV, CB, RAS, and HH wrote the paper.

## Conflict of Interest Statement

The authors declare that the research was conducted in the absence of any commercial or financial relationships that could be construed as a potential conflict of interest.

## Footnotes

^1^https://benchling.com.

^2^https://tide.nki.nl/.
