# Supplementary material for: The Human-Associated Archaeon Methanosphaera stadtmanae Is Recognized through Its RNA and Induces TLR8-Dependent NLRP3 Inflammasome Activation
Source: Front Immunol. 2017 Nov 13;8:1535. doi: 10.3389/fimmu.2017.01535 (PMC5694038; doi:10.3389/fimmu.2017.01535)
Supplement: Supplementary file 1 [file Data_Sheet_1.PDF]

Supplementary Material

The Human-Associated Archaeon *Methanosphaera Stadtmanae* is Recognized by Its RNA and Induces TLR8-Dependent NLRP3 Inflammasome Activation

Tim Vierbuchen, Corinna Bang, Hanna Rosigkeit, Ruth A. Schmitz, Holger Heine*

*** Correspondence:** Holger Heine: hheine@fz-borstel.de

1 Supplementary Figures

1.1 Supplementary Figure 1

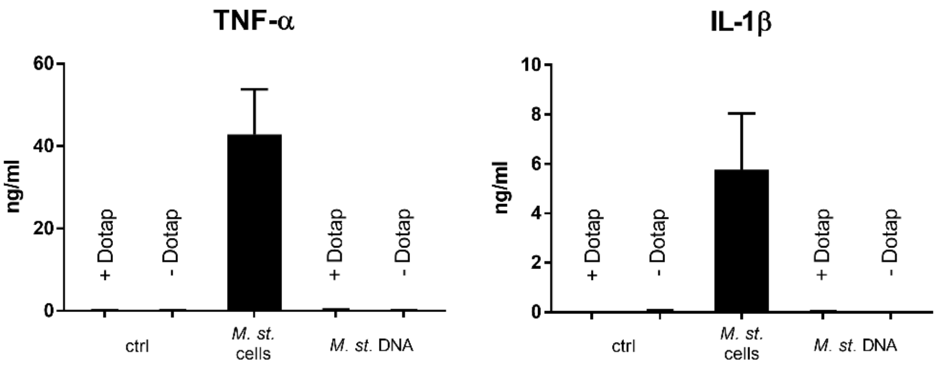

Figure S1. ***M. stadtmanae*** **DNA does not induce an immune response in human PBMCs.**

Quantification of TNF-α (left) and IL-1β (right) by ELISA of the supernatant of PBMCs either left unstimulated, stimulated by *M. stadtmanae* cells, or by purified DNA. DNA was complexed to the liposomal transfection reagent DOTAP and added to human PBMCs for 18 h. The data are from three different donors (n = 3) and shown as the mean ± SEM.

1.2 Supplementary Figure 2

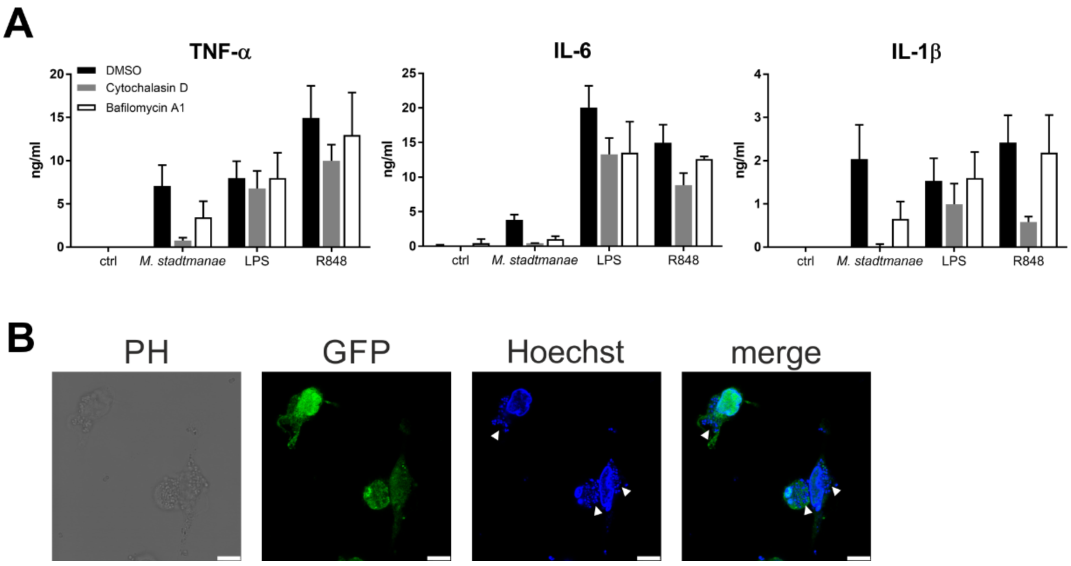

Figure S2. Response of BLaER1 monocytes to *M. stadtmanae*.

(A) Quantification of TNF-α, IL-6, and IL-1β in the supernatant of BLaER1 monocytes either left unstimulated or stimulated with 10^7 *M. stadtmanae* cells, 50 ng/ml LPS or 5 µg/ml R848 using ELISA. Cytochalasin D and Bafilomycin A (inhibitors of phagocytosis and endosomal acidification, respectively) were added 1 h prior to stimulation. The data from three independent experiments (n = 3) are shown as the mean ± SEM. **(B)** Confocal microscopy of BLaER1 monocytes 4 h after the addition of *M. stadtmanae*. GFP expression is shown in green and Hoechst 33342 staining in blue. White arrows indicate the points where *M. stadtmanae* is phagocytosed by BLaER1 cells. Scale bars: 10 µM. The images shown are representative examples from one of two independent experiments (n = 2).

1.3 Supplementary Figure 3

A

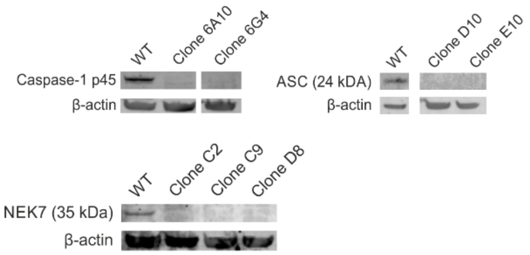

B

KO	Clone	InDel
MyD88$^{-/-}$	A8	-4/+5
	D2	+5/+14
TRIF$^{-/-}$	A1	+2/+2
	B10	+1/+1
UNC93B1$^{-/-}$	D8	-13/-2
	F7	+4/+4
TLR7$^{-/-}$	C10	+5/+5
	D4	+2/+4
TLR8$^{-/-}$	A3	-23/-2
	A9	+2/+4
TLR7$^{-/-}$xTLR8$^{-/-}$	C10A7	+5/+5 x +7/+7
	C10B12	+5/+5 x -13/+10
NLRP3$^{-/-}$	C1	+2/+4
ASC$^{-/-}$	D10	+5/+5
	E10	+4/+5
CASP1$^{-/-}$	6A10	+2/+2
	6G4	+2/+4
NEK7$^{-/-}$	C2	+2/+2
	C9	+1/+4
	D8	-1/+2
TREML4$^{-/-}$	A2	+2/+10
	D1	+5/+5

Figure S3. Generation of BLaER1 KO cell lines.

(A) Immunoblotting of BLaER1 KO cell lines was performed if specific antibodies were available. β-actin was used as the loading control. **(B)** Types of InDel mutations that were identified by TIDE analysis for all KO clones used in this study. All clones show frameshift mutations on both alleles.

1.4 Supplementary Figure 4

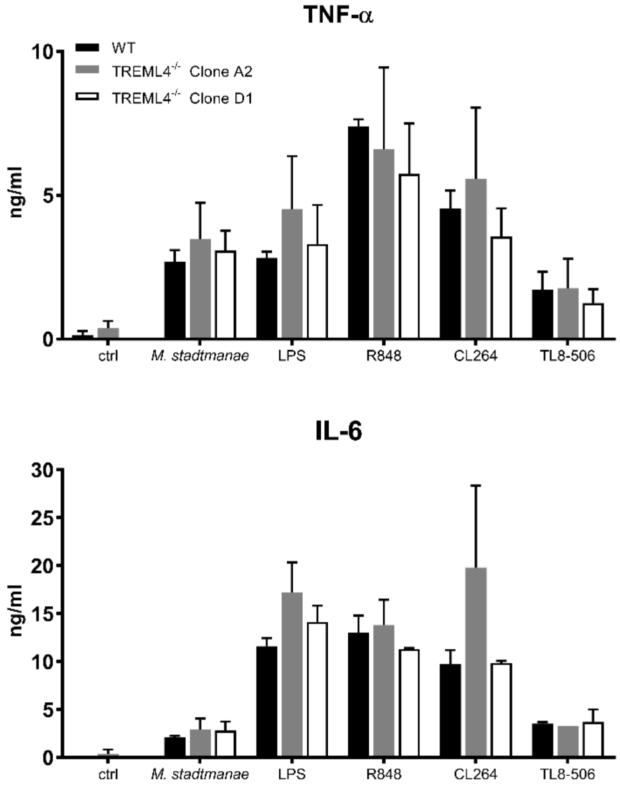

Figure S4. Response of BLaER1 TREML4$^{-/-}$ to *M. stadtmanae* and TLR7/8 agonists.

Quantification of TNF-α and IL-6 in the supernatant of BLaER1 WT and TREML4$^{-/-}$ monocytes stimulated with 10^7 *M. stadtmanae* cells, 50 ng/ml LPS or 5 µg/ml R848, CL264 or TL8-506 for 18 h using ELISA. The data from two independent experiments (n = 2) and two different knockout clones are shown as the mean ± SEM.

1.5 Supplementary Figure 5

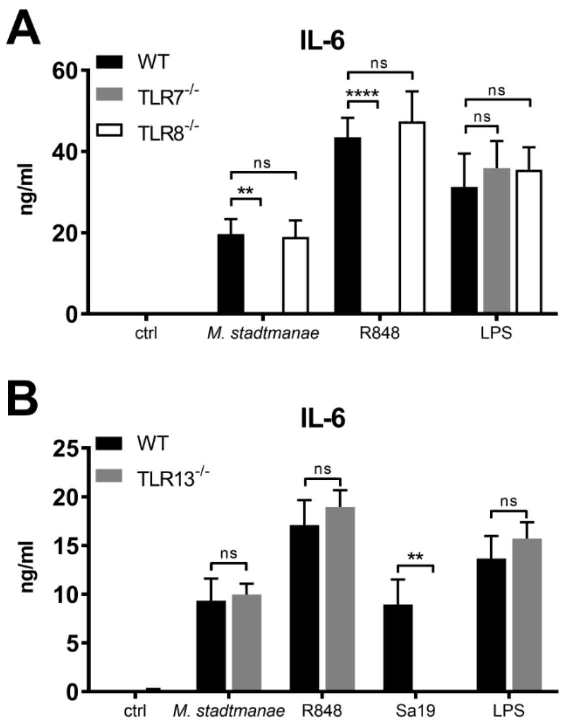

Figure S5. Stimulation of IL-6 secretion in response to *M. stadtmanae* is dependent on mTLR7 but not on mTLR8/mTLR13 in murine bone marrow-derived dendritic cells (BMDCs).

(A) Quantification of IL-6 in the supernatant of wild-type (WT), $TLR7^{-/-}$ or $TLR8^{-/-}$ murine BMDCs stimulated for 18 h with 10^7 cells of *M. stadtmanae*, 50 ng/ml LPS, 5 µg/ml R848 as measured by ELISA. **(B)** Quantification of IL-6 in the supernatant of WT or $TLR13^{-/-}$ murine BMDCs stimulated for 18 h with 10^7 cells of *M. stadtmanae*, 50 ng/ml LPS, 5 µg/ml R848 or 5 µM Sa19 (TLR13 agonist) was measured by ELISA. **(A, B)** ns: not significant, ** $P \leq 0.01$, **** $P \leq 0.00001$ (two-way ANOVA with Bonferroni *post hoc* test). The data from two to four independent experiments (n = 2-4) are shown as the mean ± SEM.

1.6 Supplementary Figure 6

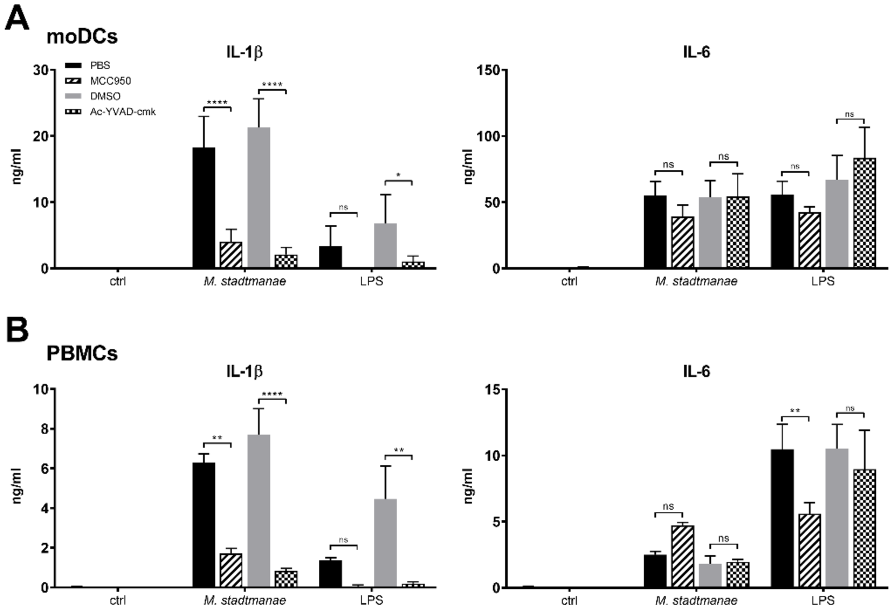

Figure S6. Secretion of IL-1β in human moDCs and PBMCs is dependent on NLRP3 and Caspase-1.

(A, B) Secretion of IL-6 (left) and IL-1β (right) in the supernatants of stimulated human moDCs **(A)** and PBMCs **(B)** was measured using ELISA. Cells were treated with either MCC950 or Ac-YVAD-cmk (inhibitors of NLRP3 and caspase-1, respectively), or control substances (medium and DMSO, respectively) 1 h prior to stimulation with 10^7 cells of *M. stadtmanae* or 50 ng/ml LPS for 18 h. ns: not significant, * $P \leq 0.05$, ** $P \leq 0.01$, *** $P \leq 0.001$, **** $P \leq 0.0001$ (repeated measures two-way ANOVA with Tukey *post hoc* test). The data from at least three different donors (n = 3-5) are shown as the mean ± SEM.

2 Supplementary Tables

2.1 Table S1. Guide RNA sequences

Gene	gRNA sequence (5‘-3‘) + PAM
ASC	ATGGACAGCAGCGCGCCCCG **CGG**
CASP1	ACAGACAAGGGTGCTGAACA **AGG**
MyD88	ACCACACTTGATGACCCCCT **GGG**
Nek7	TGTGGTTGGAACTGAGGAAC **AGG**
NLRP3	GTCTTCCTGGCATATCACAG **TGG**
TLR7	ACTGTGTACCTATTCCACTG **GGG**
TLR8 (double KO)	CGTTCTGGTGCTGTACATTG **GGG**
TLR8 (single KO)	ACAGGAAGTTCCCCAAACGG **TGG**
TREML4 A2	AAGAGTCCACATAGGAGACG **TGG**
TREML4 D1	AGCCAGGGAAGAGTCCACAT **AGG**
TRIF	GATGAGGCCCGAAACCGGTGT **GGG**
UNC93B1	CTACGACGAGACCTACCGCG **AGG**

2.2 Table S2. Primer for qRT-PCR

Gene	Name	Sequence (5‘-3‘)	Reference
IFNL1 (IFN-λ1)	huIFN-L1-fw	CGCCTTGGAAGAGTCACTCA	(Ank et al., 2006)
	huIFN-L1-rv	GAAGCCTCAGGTCCCAATTC	
IFNA14 (IFN-α14)	huIFN-a14-fw	AGGAGGAATTTGATGGCAAC	(Hillyer et al., 2012)
	huIFN-a14-rv	AGCAGCAGATGAGTTCTTTG	
IFNB1 (IFN-β)	huIFN-b-fw	AAGCAGCAATTTTCAGTGTCAG	
	huIFN-b-rv	CCTCAGGGATGTCAAAGTTCA	
HPRT	huHPRT_fw	GTCAGGCAGTATAATCCAAAGA	
	huHPRT_rv	GGGCATATCCTACAACAAACT	

3 Supplementary Methods

Stimulation of murine BMDCs

Bone marrow cells from C57BL/6 WT, TLR7$^{-/-}$, TLR8$^{-/-}$ (both are a kind gift from Douglas Golenbock, UMASS, Worcester, USA), or TLR13$^{-/-}$ (a gift from Carsten Kirschning, University of Duisburg-Essen, Essen, Germany) were differentiated into BMDCs as described previously (Stein et al., 2017). Cells were seeded at 1×10^5 cells/ml in a 96-well plate and stimulated for 18 h with 10^7 cells of *M. stadtmanae,* 50 ng/ml LPS, 5 µg/ml R848, or 5 µM Sa19 (both from Invivogen).

4 References

Ank, N., West, H., Bartholdy, C., Eriksson, K., Thomsen, A.R., and Paludan, S.R. (2006). Lambda interferon (IFN-lambda), a type III IFN, is induced by viruses and IFNs and displays potent antiviral activity against select virus infections in vivo. *J Virol* 80(9)**,** 4501-4509. doi: 10.1128/jvi.80.9.4501-4509.2006.

Hillyer, P., Mane, V.P., Schramm, L.M., Puig, M., Verthelyi, D., Chen, A., et al. (2012). Expression profiles of human interferon-alpha and interferon-lambda subtypes are ligand- and cell-

dependent. *Immunol Cell Biol* 90(8), 774-783. doi: http://www.nature.com/icb/journal/v90/n8/suppinfo/icb2011109s1.html.

Stein, K., Brand, S., Jenckel, A., Sigmund, A., Chen, Z.J., Kirschning, C.J., et al. (2017). Endosomal recognition of Lactococcus lactis G121 and its RNA by dendritic cells is key to its allergy-protective effects. *J Allergy Clin Immunol* 139(2), 667-678.e665. doi: 10.1016/j.jaci.2016.06.018.
